# The nuclear factor CECR2 promotes somatic cell reprogramming by reorganizing the chromatin structure

**DOI:** 10.1074/jbc.RA120.014598

**Published:** 2020-11-23

**Authors:** Linlin Wu, Guoqing Zhao, Shuyang Xu, Junqi Kuang, Jin Ming, Guangmin Wu, Tao Wang, Bo Wang, Ping Zhu, Duanqing Pei, Jing Liu

**Affiliations:** 1 School of Life Sciences, University of Science and Technology of China, Hefei, China; 2 CAS Key Laboratory of Regenerative Biology, South China Institutes for Stem Cell Biology and Regenerative Medicine, Guangzhou Institutes of Biomedicine and Health, Chinese Academic and Sciences, Guangzhou, China; 3 Guangdong Provincial Key Laboratory of Stem Cell and Regenerative Medicine, South China Stem Cell and Regenerative Medicine, Guangzhou Institutes of Biomedicine and Health, Chinese Academy of Sciences, Guangzhou, China; 4 Joint School of Life Science, Guangzhou Medical University-Guangzhou Institutes of Biomedicine and Health, Chinese Academy of Sciences, Guangzhou, China; 5 Bioland Laboratory (Guangzhou Regenerative Medicine and Health Guangdong Laboratory), Guangzhou, China; 6 University of Chinese Academy of Science, Beijing, China; 7 Guangdong Cardiovascular Institute, Guangdong Provincial People's Hospital, Guangdong Academy of Medical Sciences, Guangzhou, Guangdong, China

**Keywords:** SALL4, CECR2, reprogramming, DDT domain, chromatin remodeling, DMEM, Dulbecco's modified Eagle's medium, FBS, fetal bovine serum, GO, Gene Ontology, iPSC, induced pluripotent stem cell, OKSM, Oct4/Sox2/Klf4/c-Myc, PCA, Principal component analysis

## Abstract

Somatic cells can be reprogrammed into pluripotent stem cells with a minimal set of defined factors, Oct3/4, Sox2, Klf4, and c-Myc, also known as OKSM, although this reprogramming is somewhat inefficient. Recent work has identified other nuclear factors, including SALL4, that can synergize with the OSK factors to improve reprogramming dynamics, but the specific role of each of these factors remains poorly understood. In this study, we sought to learn more about the role of SALL4. We observed that SALL4 was the most significant factor in promoting OKS-induced reprogramming. To look for molecules downstream of SALL4, we screened a set of putative targets to determine whether they could promote OKS-induced reprogramming. We identified CECR2, a multidomain nuclear factor and histone acetyl-lysine reader, as a SALL4 effector. Mechanistically, we determined that SALL4 activates Cecr2 expression by directly binding to its promotor region. CECR2 in turn promotes reprogramming by forming a chromatin remodeling complex; this complex contained the SWI/SNF family member SMARCA1 and was dependent on CECR2’s DTT domain. In combination, our findings suggest that CECR2 is a novel reprogramming factor and works through a protein network to overcome epigenetic barriers during reprogramming.

Somatic cells can be reprogramed into pluripotent stem cells by overexpression of a set of nuclear factors Oct4/Sox2/Klf4/c-Myc (OKSM) or Yamanaka factors (1) in mouse, or Oct4/Sox2/Nanog/Lin28 in human (2), alternatively. This revolutionary technic, which was termed induced pluripotent stem cells (iPSCs), promised a great opportunity in regeneration medicine. Previously, the reprogramming by OKSM was low in efficiency and dynamics, and the use of oncogene c-Myc, raising the concern of tumorigenicity for the resulting iPSCs. Thus, the alteration of the reprogramming factors, especially the use of nononcogenes or other non-Yamanaka factors, will offer us a safer somatic cell reprogramming technique and new insight(s) for the underlying mechanism in somatic cell reprogramming. Indeed, a set of nuclear factors were reported to play roles in iPSC induction by replacing or combining with the Yamanaka factors. Generally, these nuclear factors can be categorized into two groups: (1) transcriptional factors, which can facilitate somatic cell reprogramming by binding to specific nuclear sequences or motifs, such as *Glis1* (3), *Nr5a2* (4), *Sall4* (5, 6), *Esrrb* (7), *Dax1* (8), *Zscan4* (9), *Tbx3* (10), and *Prdm14* (11); (2) epigenetic regulators, which facilitate somatic cell reprogramming by altering the chromatin structure or DNA/histone modifications, such as *Tet1* (12, 13), *Brg1* (14). Despite the discovery of a set of nuclear factors that facilitate reprogramming, the systematic comparison of their effects, especially in efficiency and dynamics, on reprogramming is lacking. A function ranking of these factors on reprogramming may help us to diagnose the key points of the underlying mechanisms systematically and comprehensively.

Previously, we reported a combination of seven factors reprogramming cocktails (*Nanog-Esrrb-Glis1-Jdp2-Kdm2b-Sall4-Mkk6*) (15), in which the dropout of Sall4 led a max reduction in the reprogramming efficiency, suggesting an outstanding role for Sall4 in cell fate determination. Consistently, Sall4 is reported to play an important role in a range of biological processes, such as somatic cell reprograming (5), tumorigenesis, and early embryonic development (16). However, the underlying mechanism for such an important role remains unclear. In this study, by comparing the efficiency and dynamics of a set of nuclear factors on somatic cell reprogramming, we confirm the critical role of *Sall4* on somatic cell reprogramming and identified that a new factor Cecr2, a histone acetyl-lysine reader, can promote the efficiency of somatic cell reprogramming as an effect of Sall4, attempting to improve our understanding of the epigenetic mechanisms that regulate cell fate transition.

## Results

### Sall4 promotes OSK reprogramming

**Figure 1:**
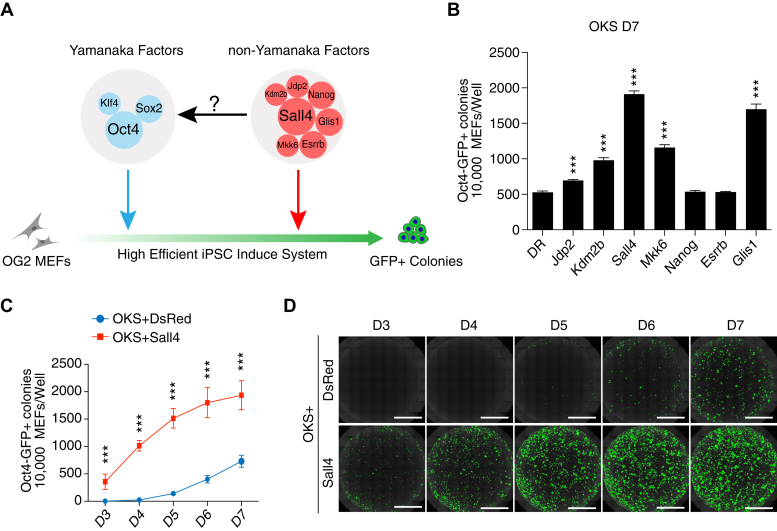
**Comparison of reprogramming efficiency for a group of nuclear factors.***A*, schematics for factors induced somatic cell reprogramming. *B*, Oct4-GFP+ colonies induced by indicated factors cultured in iCD1 culture medium at day 7. OKS: Oct4(O), Klf4(K), Sox2(S). (n = 6 wells from three independent experiments; mean ± SD, two-tailed, unpaired *t* test; ∗∗∗*p* < 0.0001). *C*, Oct4-GFP^+^ colonies induced by OKS+DsRed/OKS+Sall4 in iCD1 culture medium. (n = 6 wells from three independent experiments; mean ± SD, two-tailed, unpaired *t* test; ∗∗∗*p* < 0.0001). *D*, images for (*C*). The scale bars represent 5 mm.

Previously, we have reported a group of 7F factors (*Nanog-Esrrb-Glis1-Jdp2-Kdm2b-Sall4-Mkk6*) that can reprogram mouse fibroblasts into pluripotent stem cells with a ∼10% efficiency (15). In order to see any synergistic or cumulative effect(s) with the classic Yamanaka factors, we performed reprogramming experiments by adding each of the seven factors into Oct4, Sox2, Klf4 (Fig. 1*A*), and showed that five of the seven factors promote OKS-induced reprogramming (Fig. 1*B* and Fig. S1, *A*–*B*). Notably, Sall4, a member of the spalt-like family members (16), is the most powerful one among them, in agreement with earlier works (17, 18). We then further examined the reprogramming dynamics for Sall4 in the context of OKS-induced reprogramming with DsRed as Control and showed that Sall4 could promote iPS cell generation as early as day 3 when no iPSC colonies appear in the control group, and finally achieved ∼16% efficiency at day 7, comparing with 7% reprogramming efficiency in the control group (Fig. 1, *C*–*D* and Fig. S1, *C*–*E*).

### Sall4 reinforces reprogramming by opening and closing unique chromatin loci

**Figure 2:**
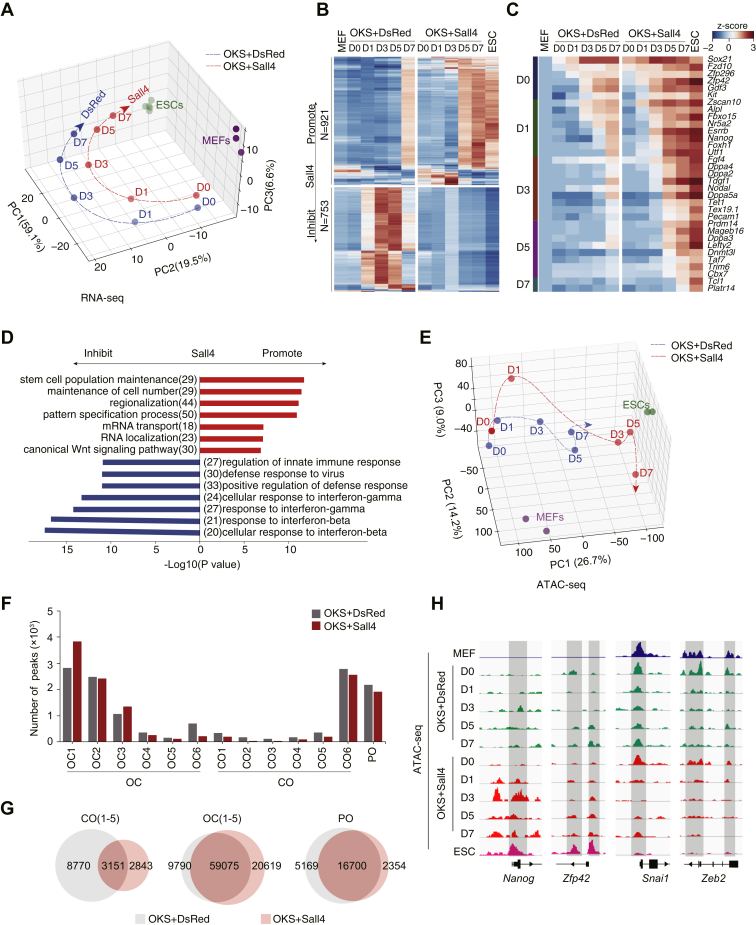
**Sall4 enhanced reprogramming through reorganizing chromatin structure.***A*, PCA analysis for RNA-seq data from OKS+DsRed/Sall4. RNA-seq data were collected from two independent experiments and merged when analyzed. *Blue* or *red fine dotted line* indicated OKS+DsRed or OKS+Sall4 reprogramming path, respectively. *B*, heatmap profile from RNA-seq analysis classified genes regulated by Sall4 in reprogramming. *C*, heatmap for the expression of stem cell–related genes in OKS+DsRed/OKS+Sall4 samples in RNA-seq data. *D*, gO analysis for Sall4 promoted or inhibited genes in (*B*). *E*, PCA analysis for ATAC-seq data from OKS+DsRed/Sall4. ATAC-seq data were collected from one independent experiment. *Blue* and *red fine dotted line* indicated OKS+DsRed and OKS+Sall4 reprogramming path, respectively. D0 (day 0), D1 (day1), D3 (day 3), D5 (day 5), D7 (day 7). *F*, number of the peaks for each CO (close–open), OC (open–close), and PO (permanently open) subgroup. *G*, number of the peaks for each CO, OC, and PO groups between OKS+DsRed and OKS+Sall4. *H*, ATAC-seq analysis showed Sall4 affected close–open and open–close peaks at representative gene site.

To reveal any new insight or mechanism for Sall4 in the process of somatic cell reprogramming, we performed time-lapse RNA-seq for OKS+Sall4– or OKS+DsRed–induced reprogramming at day 0, day 1, day 3, day 5, and day 7, respectively. Principal component analysis (PCA) for RNA-seq data showed a similar but distinct path from mouse embryonic fibroblasts (MEFs) to embryonic stem cells (ESCs) (Fig. 2*A*), with an end point at day 7 more closer to ESCs in OKS-Sall4 than in OKS-DsRed, consistent with the fact that the pluripotent genes such as *Nanog*, *Esrrb*, and *Dppa3* were expressed much higher in OKS-Sall4 samples than in OKS-DsRed samples (Fig. S2, *A*–*B*). More importantly, we showed that 921 genes are upregulated and 753 genes are downregulated by Sall4 overexpression, respectively (Fig. 2*B*). We further showed the sequential activation of the key pluripotent genes by heatmap in a day-by-day manner (Fig. 2*C*). It is quite clear that the pluripotent genes are activated faster in the OKS+Sall4 group than in the OKS+DsRed group. Consistently, among the GO terms derived from Sall4 upregulated genes are terms such as stem cell population maintenance, maintenance of cell number, and regionalization, whereas among the GO terms derived from the Sall4 downregulated genes are terms such as cellular response to interferon-beta and positive regulation of defense response (Fig. 2*D*). We then performed ATAC-seq and showed by PCA analysis that, similar to RNA-seq, OKS+Sall4 modulates the chromatin structure toward an ESC-like state more quickly than OKS+DsRed (Fig. 2*E*). We further investigated the chromatin accessibility dynamics as we described previously (Fig. 2*F*) (17). We show that, in general, the total number of OC, CO, or PO peaks are quite similar, with 3151 CO peaks, 59,075 OC peaks, and 16,700 PO peaks shared under both conditions (Fig. 2*G*). Specifically, we found that Sall4 opens the loci of pluripotent genes such as *Nanog* and *Zfp42* and closes those of fibroblast/mesenchymal genes such as *Snai1* and *Zeb2*, suggesting the important role for sall4 in activating and silencing critical genes (Fig. 2*H*).

### Cecr2 as a downstream effector of Sall4

To further investigate the downstream effector(s) of Sall4 for promoting reprogramming, we first reanalyzed the time-course RNA-Seq data for the 7F factors-induced reprogramming we reported previously (15). By comparing the gene expression profiles, we generated a panel of candidate genes regulated by Sall4 (Fig. S3*A*). GO analysis showed that genes responsible for stem cell population maintenance, stem cell differentiation, and stem cell proliferation are upregulated by Sall4 in 7F-induced reprogramming (Fig. S3*B*). We then compared the expression of stem cell–related genes in both OKS+Sall4– and 7F-induced reprogramming system and showed by Venn diagram for the common or specific genes regulated by Sall4 between the two systems (Fig. S3*C*, Table S3). To this end, we identified nine genes, *Tfcp2l1*, *Nup210*, *Lin28a*, *Cecr2*, *Trh*, *Dppa5a*, *Hmgb2*, *Rcor2*, and *Tdh*, for further functional analysis based on their relevant expression to Sall4 over-expression in 7F-induced reprogramming system (Fig. S3, *D*–*E*).

**Figure 3:**
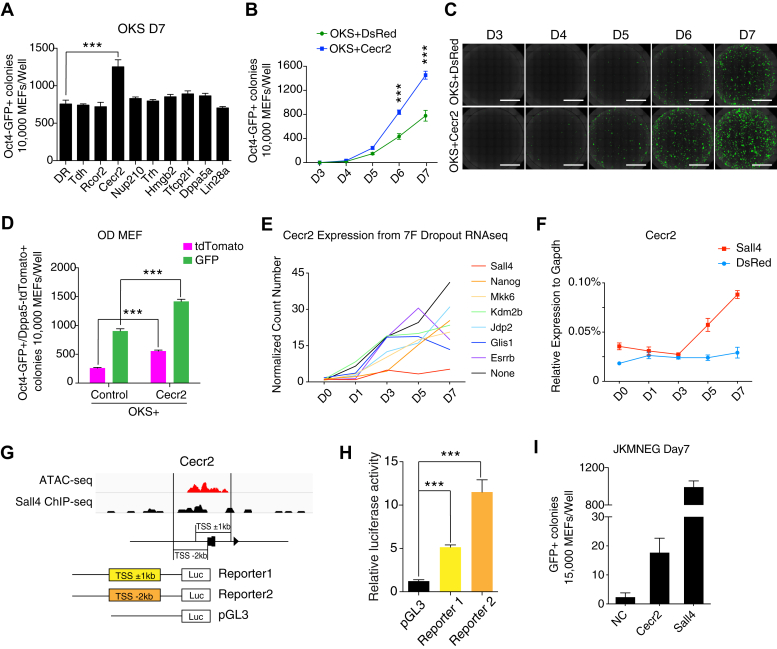
**Cecr2 promotes somatic cell reprogramming.***A*, reprogramming efficiency for indicated genes in OKS induced reprogramming at day 7 (n = 6 wells from three independent experiments; mean ± SD, two-tailed, unpaired *t* test; ∗∗∗*p* < 0.0001). *B*, numbers of Oct4-GFP+ colonies induced by OKS+DsRed or OKS+Cecr2 from 10,000 OG2-MEFs in iCD1.(n = 6 wells from three independent experiments). *C*, images for (*B*). The scale bars represent 5 mm. *D*, numbers of Oct4-GFP+ or Dppa5-tdTomato+ colonies induced by OKS+DsRed or OKS+Cecr2 from 10,000 OD-MEFs in iCD1 culture at day 7(n = 6 wells from three independent experiments; mean ± SD, two-tailed, unpaired *t* test; ∗∗∗*p* < 0.0001). *E*, expression level of Cecr2 in 7F or 7F-dropout RNA-seq data. *F*, qPCR analysis for the expression of Sall4 in OKS+DsRed or OKS+Cecr2 samples. *G*, schematic representation of the reporter designed according to the regulation of Cecr2 from mESC ATAC-seq and mESC Sall4 ChIP-seq. The sequences were cloned into pGL3 Vector (TSS, Transcription Start Sites; Luc, luciferase). *H*, Sall4 promotes the activity of Cecr2 reporter. pGL3-based reporters and TK-Renilla were cotransfected with Sall4 or control plasmid into the 293T cell line. Luciferase activity was detected at 48 h post transfection (n = 2 biological replicates each with 2 technical replicates; mean ± SD, two-tailed, unpaired *t* test; ∗∗∗*p* < 0.0001). *I*, reprogramming efficiency for Cecr2 and Sall4 in JKMNEG-based reprogramming at day 7. JKMNEG:Jdp2-Kdm2B-Mkk6-Nanog-Esrrb-Glis1. (n = 6 wells from three independent experiments).

We then over-expressed these nine genes in the OKS-induced reprogramming system and found that Cecr2 is the only hit that could significantly promote somatic cell reprogramming (Fig. 3*A*, Fig. S3*F*). We further measured the dynamics of iPSC colonies generated by Cecr2 with Oct4-GFP reporter MEFs and showed that Cecr2 mainly promoted reprogramming at the late stage of reprograming (Fig. 3, *B*–*C*). To further confirm these observations, we use Oct4-GFP/Dppa5a-Tdtomato double reporter MEFs as the starting cells and show very similar results (Fig. 3*D* and Fig. S3*G*). Consistently, Cecr2 is not activated without Sall4 in the 7F reprogramming system (Fig. 3*E*), whereas it is activated significantly when Sall4 is over-expressed in the OKS reprogramming system (Fig. 3*F*). These data suggest that Cecr2 may be regulated by Sall4 directly. To test this further, we performed ChIP(Chromatin Immunoprecipitation)-seq experiments with Sall4 antibody in mESC (Fig. 3*G*) and detected peaks in the TSS region of Cecr2 locus where open chromatin is evident with ATAC-seq. To test the significance of these peaks, we constructed two reporters by inserting two fragments near Cecr2 TSS as illustrated and found that Sall4 can activate both constructs with luciferase activity (Fig. 3*H*), suggesting that Sall4 regulated the expression of Cecr2 by directly binding to the transcription start site(TSS) region. We further showed that Cecr2 can slightly replace Sall4 in 7F-induced reprograming functionally (Fig. 3*I*), whereas there was no synergistic effect with Sall4 in OKS-induced reprogramming (Fig. S3*H*).We further showed that iPSC colonies derived from OKS+Cecr2 were similar to ESCs in morphology (Fig. S3*I*) and RNA expression profile (Fig. S3*J*). These data suggested that Cecr2 is a downstream effector of Sall4 in somatic cell reprogramming.

### Cecr2 facilitates reprogramming by reorganizing chromatin

**Figure 4:**
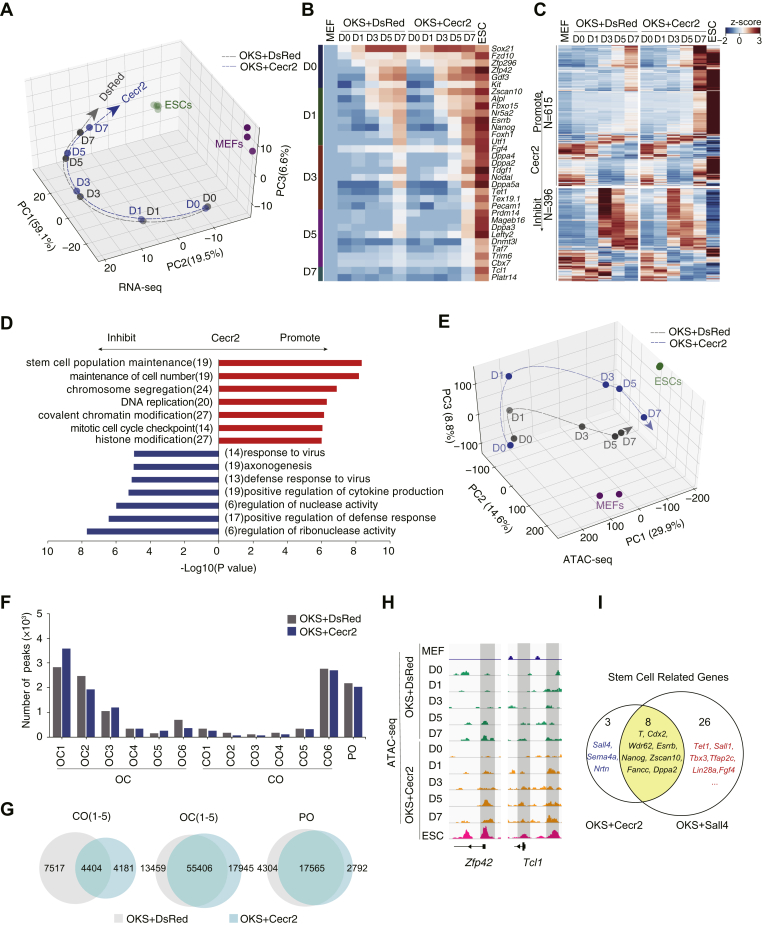
**Cecr2 reorganizes the chromatin structure at the later stage of reprogramming.***A*, PCA analysis for RNA-seq data from time course OKS+DsRed or OKS+Cecr2 samples. RNA-seq data were collected from two independent experiments and were merged when analyzed. *B*, heatmap for selected genes regulated by Cecr2 in RNA-seq dataset. *C*, heatmap of RNA-seq. *D*, heatmap for GO analysis of Cecr2 promoted or inhibited genes in (*C*). *E*, PCA analysis for ATAC-seq data from OKS+DsRed/Cecr2. ATAC-seq data were collected from one independent experiment. *Black* and *blue fine dotted line* indicated OKS+DsRed and OKS+Cecr2 reprogramming path, respectively. D0 (day 0), D1 (day 1), D3 (day 3), D5 (day 5), D7 (day 7). *F*, the total number of chromatin regions (peaks) for each CO, OC, and PO subgroup. *G*, Venn plots for the numbers of peaks for overlapping between each CO, OC, and PO groups between OKS+DsRed and OKS+Cecr2. *H*, genome view of the ATAC-seq data at *Zfp42* and *Tcl1* locus. *I*, Venn plots for the number of stem cell–related genes upregulated by OKS+Cecr2– or OKS+Sall4–induced reprogramming.

To further investigate the mechanism through which Cecr2 facilitates OKS reprogramming, we performed RNA-seq on OKS+Cecr2 and OKS+DsRed reprogramming cells at D0, D1, D3, D5, and D7. PCA analysis shows little differences between OKS+Cecr2 and OKS+DsRed samples (Fig. 4*A*). Yet, pluripotent genes, such as Fzd10, Zfp42, and Zscan10, Fbxo15, have higher expression levels at later stage when Cecr2 overexpressed (Fig. 4*B*), consistent with the higher efficiency. Furthermore, we can identify 615 genes upregulated and 396 downregulated by Cecr2, respectively (Fig. 4*C*). Gene Ontology or GO analysis reveals that genes upregulated by Cecr2 are enriched in GO terms such as maintenance of cell number, stem cell population maintenance, chromosome organization, and DNA repair, and those downregulated by Cecr2 as regulation of ribonuclease activity, axonogenesis, regulation of nuclease activity, etc. (Fig. 4*D*).

The discrepancy between the overall RNA-seq results and select gene expression in Figure 4*A* *versus* Figure 4, *B*–*C* suggests that Cecr2 may regulate only a specific set of genes. To confirm this, we performed ATAC-seq on OKS+Cecr2 and OKS+DsRed reprogramming cells at D0, D1, D3, D5, and D7. Indeed, unlike RNA-seq data, PCA analysis for ATAC-seq data demonstrates quite clear divergent paths between OKS+Cecr2 and OKS+DsRed (Fig. 4*E*). Consistent with the RNA-seq data, the total number of CO, OC, or PO peaks between the two conditions are quite similar (Fig. 4*F*), with 4404 CO peaks, 55,406 OC peaks, and 17,565 PO peaks shared in both conditions (Fig. 4*G*). However, the chromatin loci near pluripotent genes such as Zfp42 and Tcl1 are opened more quickly in the Cecr2 group than in the DsRed control group (Fig. 4*H*), suggesting that Cecr2 promoted reprogramming by reorganizing chromatin structure at late stage of reprogramming. To further investigate the similarities or differences upon the impact to the pluripotent regulation network between Cecr2 and Sall4, we compared the RNA-seq data from OKS+DsRed, OKS+Sall4, and OKS+Cecr2 by heatmap and Gene Ontology analysis and further compared them by using a set of stem cell–related genes and showed that 8 genes such as *T*, *Cdx2*, *Wdr62*, *Esrrb*, *Nanog*, *Zscan10*, *Fancc*, and *Dppa2* are regulated by both CECR2 and SALL4; 3 genes such as Sall4, Sema4a, and Nrtn are regulated by CECR2; 26 genes such as *Tet1*, *Sall1*, *Tbx3*, *Tfap2c*, *Lin28a*, and *Fgf4* are regulated by SALL4, respectively. (Fig. 4*I*, Table S3). We also compared the chromatin accessibility dynamics data from OKS+DsRed, OKS+Sall4, and OKS+Cecr2, and PCA analysis showed that the chromatin state of Cecr2 is very close to Sall4 group at the late stage of reprogramming (Fig. S4, *A*–*C*). These data suggested that CECR2 plays a significant role in reorganizing chromatin in the late stage of reprogramming.

### The DDT domain is essential for the reprogramming activity of CECR2

**Figure 5:**
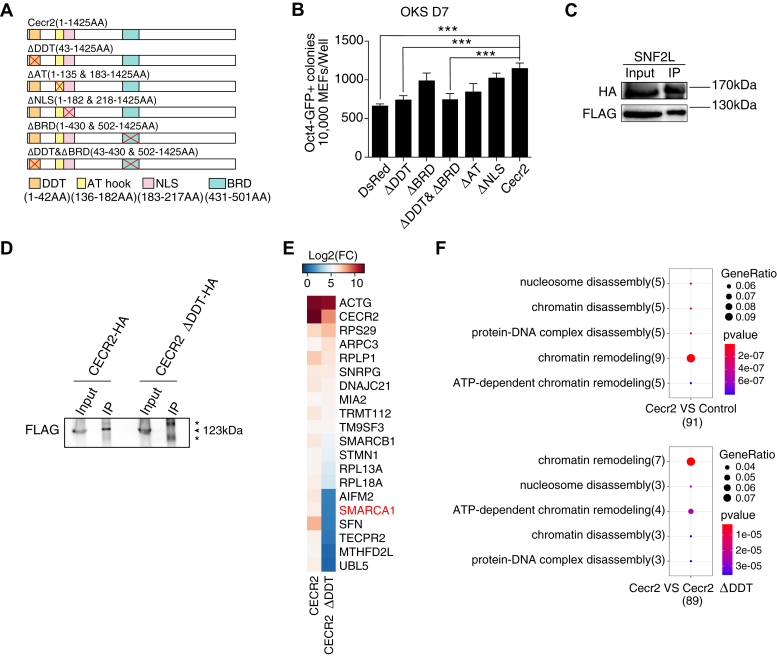
**Effect of cecr2 gene structure on its reprogramming activity.***A*, schematic illustration of Cecr2 structure and mutants. *B*, effect of various Cecr2 mutants on OKS-induced reprogramming (n = 6 wells from three independent experiments; mean ± SD, two-tailed, unpaired *t* test; ∗∗∗*p* < 0.001.). *C*, IP and immunoblot analysis of the interaction between CECR2-HA and SNF2L-FlAG. *D*, IP and immunoblot analysis of CECR2-HA/CECR2 ΔDDT-HA and SNF2L-FLAG indicates the DDT domain is essential for the interaction between CECR2 and SNF2L. SNF2L with a molecular weight of 123 kDa is indicated. Asterisks indicate non-specific bands. *E*, heatmap of IP-MS analysis showing Top 20 proteins enriched in CECR2. NOTE that the deletion of DDT domain disrupts the interaction between CECE2 and SMARCA1(SNF2L). *F*, heatmap of GO analysis for top 100 proteins enriched in OKS+Cecr2-3×FLAG VS OKS+DsRed-3×FLAG (Control) or top 100 proteins enriched in OKS+Cecr2-3×FLAG VS OKS+Cecr2 ΔDDT-3×FLAG reprogramming in day 3 through FLAG-IP MS.

CECR2 is a multidomain transcription factor (19) that may modulate chromatin remodeling through its DDT (involved in chromatin remodeling with ISWI), BRD (bromodomain, binds acetylated lysine residue), AT hook, or NLS domains separately or in combination (Fig. 5*A*). To see which one is responsible for enhancing reprogramming, we generated a set of constructs as shown in Figure 5*A* and show that the deletion of the DDT domain results in loss of ability to promote OKS reprogramming (Fig. 5*B*). Previously, Cecr2 was reported to remodel the chromatin structure by forming a complex with SNF2L(SMARCA1) (18)，a member of the ISWI family of protein. By co-overexpressing CECR2-HA and SMARCA-3×FLAG in 293 cells, we confirmed this interaction by coimmunoprecipitation (coIP) experiment (Fig. 5*C*) and further showed that the DDT domain was necessary for this protein–protein interaction (Fig. 5*D*). Furthermore, we checked this interaction by IP-MS experiment and showed by heatmap that CECR2 could enrich SMARCA1, whereas Cecr2-DTT could not (Fig. 5*E*, Fig. S5*A*). Consistently, GO analysis for CECR2-specific interaction proteins showed a significant enrichment in GO term for chromatin remodeling (Fig. 5*F*, Fig. S5*B*). These data indicated that CECR2 regulates somatic cell reprogramming by DTT domain–mediated chromatin remodeling.

### CECR2 is dispensable for pluripotency

**Figure 6:**
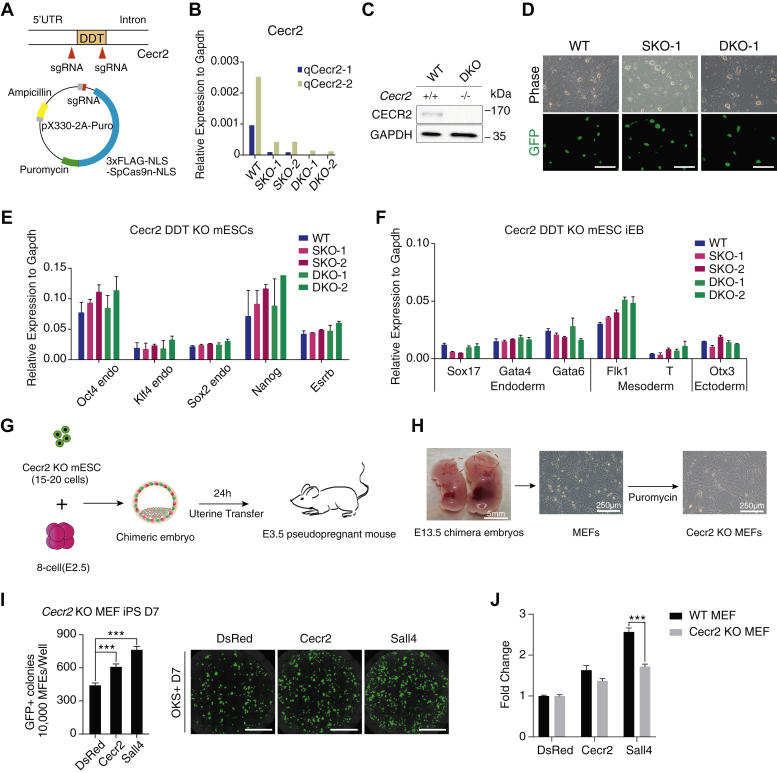
**Knockout of Cecr2 in mESCs.***A*, schematic of Cecr2 gene-targeting strategy. Two sgRNAs targeted DDT domain were transfected into mESCs with cas9. Cells were cultured feeder free in N2B27+2i+LIF(2iL) medium. *B*, qPCR analysis for the validation of Cecr2 knockout in mESCs. *C*, immunoblotting of CECR2 protein in Cecr2^+/+^ and Cecr2^−/−^ ESC in 2i+LIF medium. *D*, images of Cecr2 KO in Oct4-GFP reporter cell line under 2iL condition. (SKO, single allele knockout; DKO, double allele knockout). The scale bars represent 250 μm. *E*, relative expression of the pluripotency genes in Cecr2 WT, SKO, and DKO mESC. *F*, relative expression of the indicated genes representing early germ layer commitment during the embryonic body (EB) formation by Cecr2 WT, SKO, and DKO mESC. *G*, the work flow for the generation of Cecr2 knockout chimera embryos. *H*, chimeric embryos show the NTC exencephaly. Cecr2 knockout MEFs (Cecr2 KO OG2MEFs) were isolated from E13.5 embryos. Cecr2 knockout MEFs were selected by puromycin (2 μg/ml). *I*, numbers of Oct4-GFP+ colonies from 15,000 Cecr2 KO OG2-MEFs induced by OKS+Cecr2 or OKS+Sall4 in iCD1 culture conditions at day 7. *J*, the effect of Cecr2 knockout on OKS+Cecr2– or OKS+Sall4–induced reprogramming.

To further investigate the role Cecr2 may play in pluripotency and differentiation, we inactivated *Cecr2* by CRISPR-Cas9–mediated gene editing in mESC (Fig. 6*A*) and confirmed the inactivation of Cecr2 at RNA (Fig. 6*B*) and protein levels (Fig. 6*C*). Cecr2 single or double allele knockout ESC are very similar to WT ESC in morphology (Fig. 6*D*), expression of pluripotent genes (Fig. 6*E*), and also the three germ layer markers expression when undergoing embryoid bodies differentiation *in vitro* (Fig. 6*F*). These data demonstrate that Cecr2 is dispensable for pluripotency or early embryonic development. We further tested the development potential of double allele knockout ESC by injecting the cells into diploid or tetraploid embryos followed by a transfer to pseudo pregnant mouse (Fig. 6*G*). No live embryos were obtained at 13.5 d.p.c(days post coitum) in tetraploid injection group, whereas 10 live chimera embryos were obtained in the diploid injection group (Fig. S6*A*), with 2 of 10 showing typical neural tube defected exencephaly (Fig. 6*H*). We further purified Cere2 KO MEFs by puromycin selection for MEFs derived from E13.5 chimera embryos (Fig. 6*H*). The Cecr2 KO MEF could be reprogramed into iPSC colonies successfully at an efficiency of ∼5% (Fig. 6*I*). Consistently, knockdown of Cecr2 by shRNA shows little impact on the reprogramming efficiency (Fig. S6*B*). These data suggested that Cecr2 is not essential for iPSC generation. However, Sall4 promotes reprogramming more significantly in WT MEFs than in Cecr2 KO MEFs (Fig. 6*J*). These data suggested that SALL4 promotes somatic cell reprogramming partially by CECR2.

## Discussion

**Figure 7:**
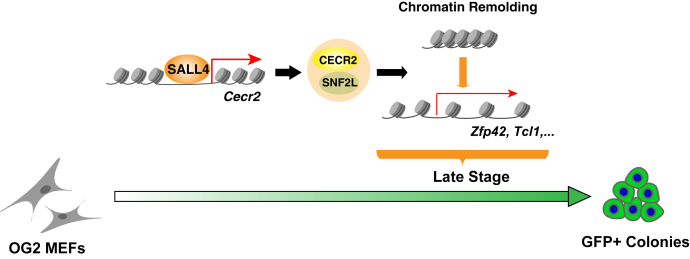
**A model for Sall4-Cecr2–mediated somatic cell reprogramming.** During somatic cell reprogramming, Cecr2 was upregulated by SALL4’s binding to its promoter region. The resulting CECR2 protein formed an SNF2L contained chromatin remodeling complex, which promotes somatic cell reprograming in the late stage.

Transcriptional factor–based somatic reprogramming is a promising tool for both the study of fundamental mechanism in cell biology and the cell-based therapies in regenerative medicine. A challenge regarding optimization and standardization of this technic is the identification of a gene set that can achieve rapid and efficient iPSC generation in a quantifiable and predictable way. Previously, we demonstrated that Sall4 is the most indispensable transcriptional factor among a new set of 7F reprogramming factor cocktails by which high-quality iPSC colonies could be achieved rapidly and efficiently (15). In this study, we first confirmed the significant role of Sall4 overexpression in the classic Yamanaka factors Oct4/Sox2/Klf4–induced somatic cell reprogramming and further investigated the chromatin accessibility and gene expression dynamics. Importantly, we identified Cecr2, a histone acetyl-lysine reader, is an important responder of SALL4 in somatic cell reprogramming. These results indicate that Cecr2 acts as an effector of Sall4 to modulate the landscape of chromatin accessibility (Fig. 7), which improved our understanding for transcription factors-induced cell fate transition in lineage specification, *trans*-differentiation, and somatic cell reprogramming.

In addition to the classic Yamanaka factors, a series of transcriptional factors have been reported to mediate somatic cell reprogramming (19). Among them, Oct4 was regarded to be the most important one, as single Oct4 alone can reprogram MEFs into the pluripotent state (20). Previously, Sall4 has been described as a “star” factor that links between stem cells, development, and cancer (16), and amounts of regulators, partners and targets of SALL4 were identified. Recently, our finding indicated that Sall4 showed an increasing importance beyond other reprogramming factors by a dropout assay in a newly setup 7F reprogramming cocktails (15). The identification of CECR2 extends the reprogramming factor family members. More importantly, CECR2 has been reported to be a member of an important family of chromatin modification complexes, and this finding will provide new insights into the mechanisms by which Sall4 regulates somatic reprogramming through epigenetic mechanisms, in particular by altering chromatin accessibility.

Chromatin modification complexes such as BAF have been reported to be involved in regulating somatic reprogramming (14). CECR2 has been reported to be involved in somatic reprogramming for the first time. Interestingly, CECR2 has been reported to form chromatin modification complex with SNF2L (20), suggesting that this complex may be involved in chromatin accessibility changes during Sall4-driven somatic reprogramming, thereby facilitating the somatic reprogramming process. It is important to note whether this CECR2-dependent SALL4-driven chromatin modification process is specific to somatic reprogramming or not. In addition, it is necessary to investigate whether there are similar mechanisms in the fate determination of other cells.

## Experimental procedures

### Cell culture

Male Oct4–GFP transgenic allele carrying mice (CBA/CaJ × C57BL/6J) were bred with 129Sv/Jae female mice to get OG2MEFs from 13.5 d.p.c mouse embryos. ODMEFs were isolated from 13.5 d.p.c mouse embryos from hybrids of Dppa5a-tdTomato reporter male mice (129) and OG2 female mice (CBA/CaJ × C57BL/6J). MEFs and PlatE cells were cultured in Dulbecco's modified Eagle's medium (DMEM) supplemented with 10% fetal bovine serum (FBS), non-essential amino acids(NEAA) and GlutaMAX supplement. mESCs were maintained on feeder layers with mES+2i medium (DMEM, 15% FBS, NEAA, GlutaMAX, β-Mercaptoethanol, PD0325901, Chir99021, LIF) or feeder free with N2B27+2i medium (knockout/DMEM, DMEM, N2, B27, NEAA, GlutaMAX, β-ME, PD0325901, Chir99021, LIF).

### Generation of iPSCs

A total of 8 × 10^6^ plat-E cells were seeded into 100-mm dish 1 day before transfection. Calcium phosphate transfection was performed when cell confluence reached 80%. Retrovirus supernatants were collected 48 and 72 h post transfection and filtered by 0.45-μm filter (Millipore). Retrovirus supernatants could be stored at room temperature for 24 h About 12 to 24 h before infection, MEFs were seeded into 12- or 24-well plate at a density of 5000 cells/cm^2^. Each retrovirus supernatant and MEFs culture medium were mixed at a ratio of 1:1 with 4 g/ml polybrene to infect MEFs. After twice infection, MEFs were changed to iCD1 medium (21) and the day was defined as day 0. GFP+ colonies and td-Tomato+ colonies at different time points were counted to indicate reprogramming efficiency.

### Generation of Cecr2 knockout OG2 mESCs

The Cecr2 knockout mESC line was generated by genome editing using CRISPR-Cas9. In brief, two pX330-puro vectors containing sgCecr2-1/2 were transfected into OG2 mESCs with lipo3000 in a ratio of 1:1. The colonies were selected by puromycin (2 μg/ml) for 3 days. The sgRNA used for genome editing and PCR primers used for knockout identification were listed in Table S1.

### Flow cytometry

Reprogramming cells were collected at different days and digested by 0.25% trypsin. Cells were suspended with flow cytometry buffer (PBS with 2% FBS). After being filtered, suspensions were analyzed with Fortessa cytometer (BD Biosciences, San Jose, CA). The flow cytometry data were analyzed using FlowJo software.

### Western blot

Western blots were performed using typical laboratory procedures with the antibodies anti-CECR2 (sc-514878, Santa Cruz Biotechnology) and anti-GAPDH (MAB374, Millipore).

### Luciferase activity analysis

The pGL3-reporters were designed according to mESCs ATAC-seq and mESCs Sall4 ChIP-seq result. The analysis result indicated that Sall4 regulated Cecr2 at the TSS site. We constructed DNA sequences TSS ± 1kb and TSS-2kb into pGL3-Basic vector. 293T cells transfected with pMX-Sall4 were planted in 24-well plates at a density of 200,000 per well, and the pGL3-Basic vector (0.5 μg per well)/pGL3-reporter (500 ng per well, pGL3-TSS ± 1kb/pGL3-TSS-2kb) were co-transfected into the cells with TK-Renilla (5 ng per well) using Lipo3000 Transfection Reagent (L3000015, Invitrogen) according to the manufacturer’s instructions. Forty-eight hours after transfection, the cells were washed with PBS and lysed in PLB (Promega), and the luciferase activity was detected according to the instructions for the Dual-Luciferase Reporter Assay System (Promega).

### Coimmunoprecipitation

Plat-E cells were transfected with pMX-Cecr2-HA/pMX-Cecr2 ΔDDT-HA and pMX-Smarca1-3×FLAG at the same time. Thirty-six hours after transfection, cells were digested, counted, and lysed. One milliliter lysis buffer (150 mM NaCl, 50 mM Tris-HCl pH 7.4, 2 mM EDTA, 1% NP-40, and protease inhibitors) was used to lyse 1 × 10^7^ cells. Cells were lysed for 30 min at 4 °C. The lysates were centrifuged (13,000*g* for 10 min) and only the supernatant was collected. Immunoprecipitation was performed by 400 μl supernatant and 20 μl anti-HA beads (88837, Thermo Scientific) for 40 min at room temperature. Beads were washed with lysis buffer for five times and then boiled in SDS loading buffer for 10 min to resuspend sample. Antibodies used for coIP were anti-HA (3724s, CST); anti-FLAG (F1804, Sigma).

### Immunoprecipitation-MS

Whole cell extracts of reprogramming cells at day 3 with Cecr2-FLAG/DsRed-FLAG overexpression (OKS+Cecr2-3×FLAG/OKS+DsRed-3×FLAG) were prepared using lysis buffer (50 mM Tris pH 8.0, 150 mM NaCl, 10% glycerol, 0.5% NP40) with freshly added Complete Protease inhibitors (Sigma, 1187358001). Cells were incubated for 2 h at 4 °C with rotation. Soluble cell lysates were collected by centrifugation (12,000*g*, 15 min at 4 °C). One milligram of cell lysates was incubated with either FLAG antibody or matched IgG overnight at 4 °C with rotation. Combined Protein A/G magnetic beads (Bio-Rad, 1614833) were added for another 1.5 h. Beads were then washed three times with wash cell lysis buffer and one time with PBS. After complete removal of PBS, immunoprecipitated proteins were digested using on-bead digestion protocol as described before (22). Briefly, beads were incubated with 100 μl of elution buffer (2 M urea, 10 mM DTT, and 100 mM Tris pH 8.5) for 20 min. Then, iodoacetamide (Sigma, I1149) was added to a final concentration of 50 mM for 10 min away from light, followed by 250 ng of trypsin (Promega, V5280) for partial digestion for 2 h. After incubation, the supernatant was collected in a separate tube. The beads were then incubated with 100 μl of elution buffer for another 5 min, and the supernatant was collected in the same tube. All these steps were performed at RT in a thermoshaker at 1500 rpm. Combined elutes were digested with 100 ng of trypsin overnight at RT. Finally, tryptic peptides were acidified to pH < 2 by adding 10 ml of 10% TFA (trifluoroacetic acid, Sigma, 1002641000) and desalted using C18 Stagetips (Sigma, 66883-U) prior to MS analyses.

### Induction of embryoid bodies *in vitro*

In order to induce embryoid bodies differentiation *in vitro*, mESCs were digested by 0.05% trypsin and cultured in suspension at a density of 1000 cells/20 μl. Suspension medium was mES medium (DMEM, 15% FBS). After 6 days of induction, embryoid bodies were collected and lysed with TRIzol to analyze germline genes expression. Germline genes include endoderm (Sall4, Sox17, Gata4, Gata6), mesoderm (Flk1, T), and ectoderm (Nestin, Sox1).

### Generation of Cecr2 knockout MEFs

In order to get Cecr2 knockout MEFs, puromycin-resistant Cecr2 knockout OG2 mESCs were incubated with one or two E2.5 embryos to form chimeric or tetraploid embryos. In brief, E2.5 embryos were treated with acid Tyrode's solution to remove zona pellucida. Then one or two embryos was incubated with 15 to 20 Cecr2 knockout OG2 mESCs in incubator for 24 h to form blastocyst, followed by implantation into pseudopregnant ICR female mice. Chimeric MEFs were isolated from E13.5 embryos. Cecr2 knockout MEFs were selected by puromycin (2 μg/ml) for the following 3 days. All of the animal experiments were performed with the approval and according to the guidelines of the Animal Care and Use Committee of the Guangzhou Institutes of Biomedicine and Health.

### RT-qPCR and RNA-Seq

Total RNA was extracted with a TRIzol-based protocol and converted into cDNAs with ReverTra Ace (Toyobo) and oligo-dT (Takara), and then analyzed by qPCR with Premix Ex Taq (Takara). Libraries were constructed according to the instructions for the Illumina TruSeq RNA Sample Prep kit (RS-122-2001, Illumina). Sequencing was performed on a MiSeq instrument with Miseq Reagent Kit V2 (MS-102-2001, Illumina). Data were analyzed with RSEM software. The qPCR primers for pluripotent genes, Germline genes, and Cecr2 expression–related genes used in this research can be found in Table S2.

### ATAC-seq

ATAC-seq was performed as previously described (23). In brief, 50,000 cells were collected and washed once with 50 ml cold PBS. Then 50 ml lysis buffer (10 mM Tris-HCl pH 7.4, 10 mM NaCl, 3 mM MgCl_2_, 0.2% (v/v) IGEPAL CA-630) was used to resuspend cells. The suspension was then centrifuged at 500*g* for 10 min at 4 °C, followed by addition of 50 ml transposition reaction mix (25 ml TD buffer, 2.5 ml Tn5 transposase, and 22.5 ml nuclease-free H_2_O) of Nextera DNA library Preparation Kit (96 samples) (FC-121-1031, Illumina). After suspension, samples were amplified by PCR and incubated at 37 °C for 30 min. DNA was isolated using a MinElute Kit (QIAGEN). ATAC-seq libraries were subjected to five cycles of preamplification first to determine the number of cycles required for the second round of PCR. Then the amplified libraries, amplified by PCR for an appropriate number of cycles, were purified with a Qiaquick PCR (QIAGEN) column. The concentration of library was measured using a KAPA Library Quantification Kit (KK4824). Library integrity was checked by gel electrophoresis. Finally, the ATAC library was sequenced on a NextSeq 500 using a NextSeq 500 High Output Kit v2 (150 cycles) (FC-404-2002, Illumina).

### ChIP-seq

Sall4 ChIP was performed with CUT&Tag (Cleavage Under Targets and Tagmentation, Hyperactive pA-Tn5 Transposase for CUT&Tag, S603-01, Vazyme) method. In brief, 60,000 mESCs were collected and bounded to Concanavalin A–coated beads. Then cells were resuspended in antibody buffer and incubated with primary (SALL4A, abcam, ab29112) and secondary antibodies in order. Then samples were incubated with pA-Tn5 transposase. After transposon activation and tagmentation, DNA was isolated, amplified, and purified to construct ChIP-seq library. The ChIP DNA library for NextSeq 500 sequencing was constructed with VAHTS Turbo DNA Library Prep Kit for Illumina (Vazyme Biotech) according to manufacturer’s instructions. AMPure XP beads were used for purification steps. The library was quantified with VAHTS Library Quantification Kit for Illumina (Vazyme Biotech). Libraries were sequenced on an Illumina NextSeq 500 v2 using 50-bp paired-end reads.

### ATAC-seq analysis

All the sequencing data were mapped onto the mm10 mouse genome assembly using the bowtie2 software. Low-quality mapped reads were removed using samtools (view –q 35) and only unique reads mapping to a single genomic location or strand were kept. We removed mitochondrial sequences using ‘grep –v ‘chrM’. Biological replicates were merged, and peaks were called using dfilter (24) (with the settings: -bs = 100 –ks = 60 –refine). BigWig files were produced by genome Coverage Bed from bedtools (scale = 107/<each_sample’s_total_unique_reads >) and then bed graph to BigWig. Gene ontology and gene expression measures were first called by collecting all transcription start sites within 10 kb of an ATAC-seq peak and then performing GO analysis with goseq (25). Other analysis was performed using glbase (26).

### RNA-seq, ChIP-seq analyses

RNA-seq clean reads were mapped to mouse transcript annotation of Gencode vM15 version on mm10 genome using RSEM (27). We chose *trans* per million value for the normalization and evaluation of gene expression levels. Meanwhile, ChIP-seq clean reads were mapped to mm10 genome using Bowtie2 package (28). Then we applied MACS2 (29) and Dfilter (24) to call the enriched peaks, then used Deeptools (30) and Homer (31) to calculate the ChIP-seq peak profiles near the gene. Data analysis and visualizations were performed in R environment.

## Data availability

ATAC-seq, RNA-seq, and ChIP-seq data that support the findings of this study have been deposited in the Gene Expression Omnibus (GEO) under accession codes GSE147678, GSE147679, and GSE147680, respectively. A super series of all datasets can be found at GSE147681. Previously published OKS and mESCs ATAC-seq data that were reanalyzed here are available under accession code GSE93029. Previously published 7F RNA-seq data that were reanalyzed here are available under accession code GSE127927. All other data supporting the findings of this study are available from the corresponding author on reasonable request.

## Conflict of interest

The authors declare that they have no conflicts of interest with the contents of this article.
