# Supplementary material for: The nuclear factor CECR2 promotes somatic cell reprogramming by reorganizing the chromatin structure
Source: J Biol Chem. 2020 Nov 23;296:100022. doi: 10.1074/jbc.RA120.014598 (PMC7948406; doi:10.1074/jbc.RA120.014598)
Supplement: Supplementary Figures and Tables [file mmc1.pdf]

Supplementary Figure 1

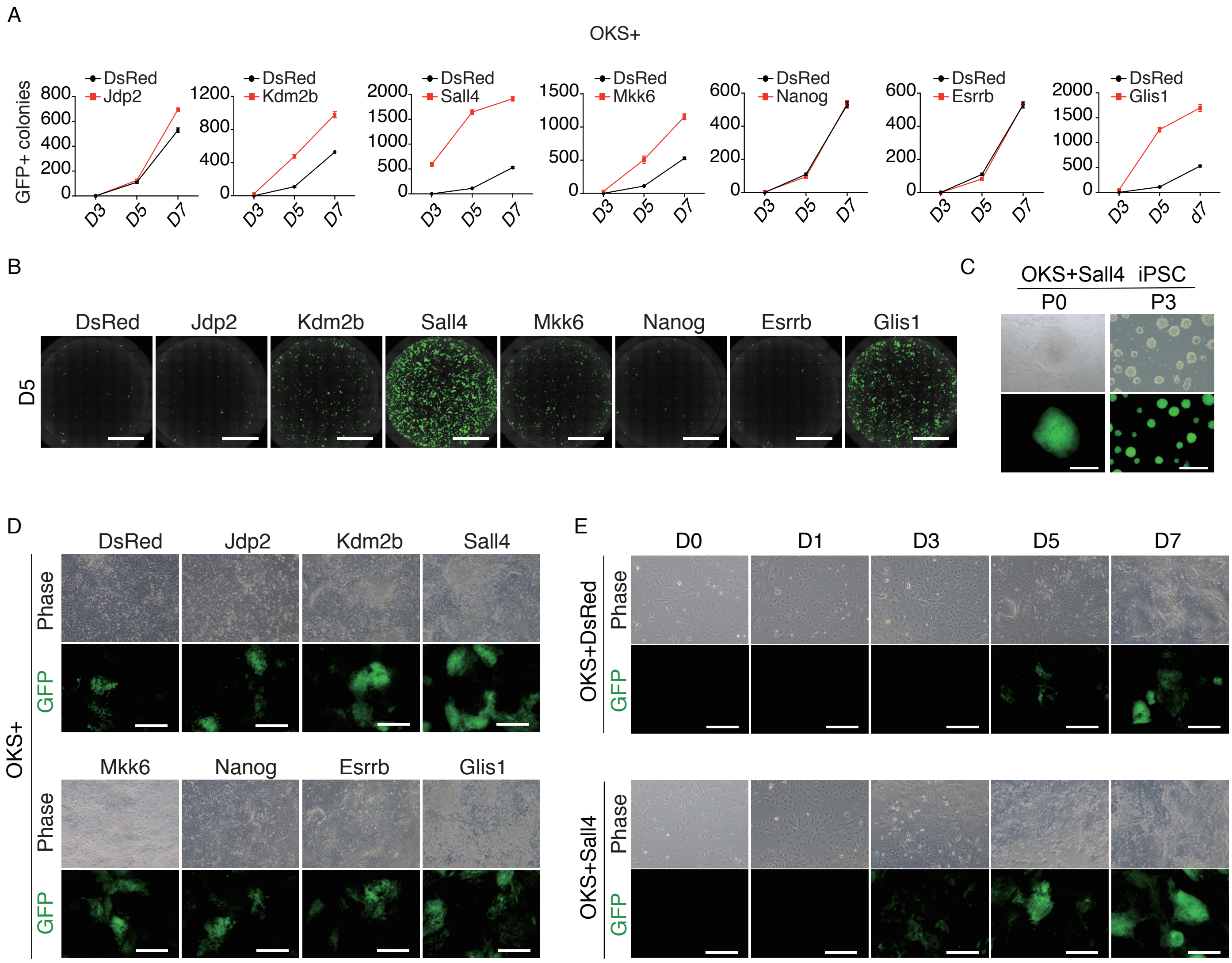

Supplementary Figure 1. Related to Figure 1.

Sall4 Enhanced OKS Induced Reprogramming.

(A)Number of time course Oct4-GFP+ colonies from 10,000 OG2MEFs infected with OKS+ DsRed/Jdp2/Kdm2b/Sall4/Mkk6/Nanog/Esrrb/Glis1 in iCD1 culture conditions in 7 days(n=6 wells from three independent experiments).

(B)Images for (A) at day5. Scale bars, 5mm.

(C) Morphology of iPSCs from OKS+Sall4 treatment. Scale bars, 250 µm.

(D) Images for Figure 1B. Scale bars, 250 µm.

(E)Representative images of the OG2MEF reprogramming process at different time points. Scale bars, 250 µm.

Supplementary Figure 2

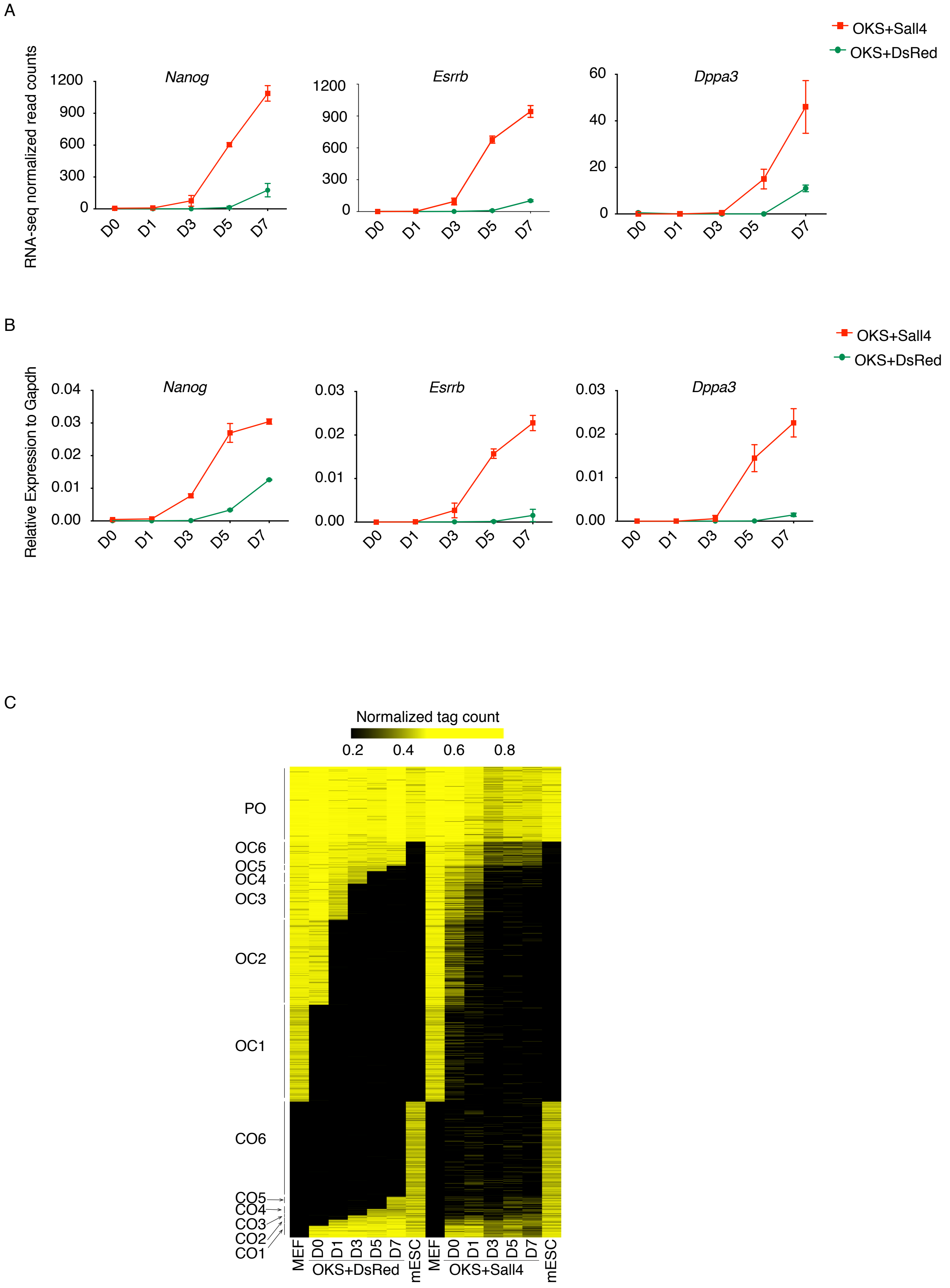

Supplementary Figure 2. Related to Figure 2.

The overexpression of Sall4 in OKS affects pluripotent genes expression and chromatin state.

(A)The expression level of genes in Figure 2C from RNA-seq data. Pluripotency related genes were upregulated in OKS+Sall4 reprogramming.

(B) qPCR analysis of the expression of genes in (A).

(C)CADs for OKS+DsRed and OKS+Sall4. CO, Close-Open; OC, Open-Close; PO, Permanently Open; OC6(OCF), open during but close in ESCs; CO6(COF), closed during but open in ESCs.

Supplementary Figure 3

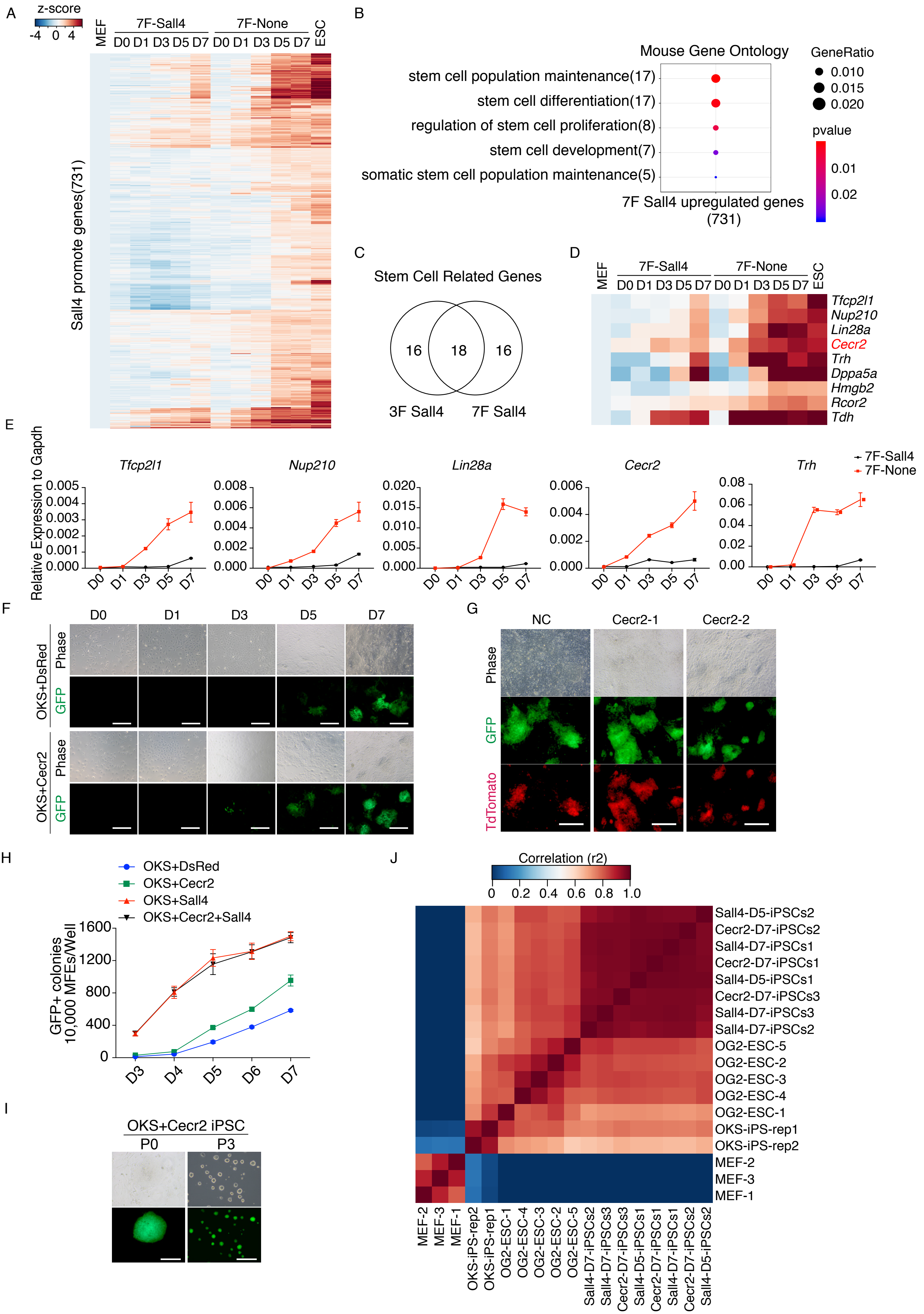

Supplementary Figure 3. Related to Figure 3.

Sall4's downstream gene Cecr2 could enhance OKS induced reprogramming.

(A) Heatmap for the RNA-seq data of 7F and 7F-Sall4 showed Sall4 upregulated genes.

(B) Gene Ontology (GO) analysis for genes upregulated mediated by Sall4 during 7F reprogramming.

(C) Stem cell releated genes upregulated by Sall4 during OKS+Sall4 or 7F reprogramming.

(D) The expression level of Sall4's downstream gene in 7F reprograming.

(E) qPCR analysis of the expression of genes in (D).

(F) Representative images of the OG2MEF reprogramming process at different time points. Scale bars, 250 µm.

(G) Images of Reprogrammed OD MEF in day7. Scale bars, 250 µm.

(H) The overexpression of Cecr2 in OKS+Sall4 couldn't further improve reprogramming efficiency.

(I) Morphology of iPSCs from OKS+Sall4 treatment. Scale bars, 250 µm.

(J) The R2 correlation coefficient matrix of all versus all RNA-seq datasets as indicated.

Supplementary Figure 4

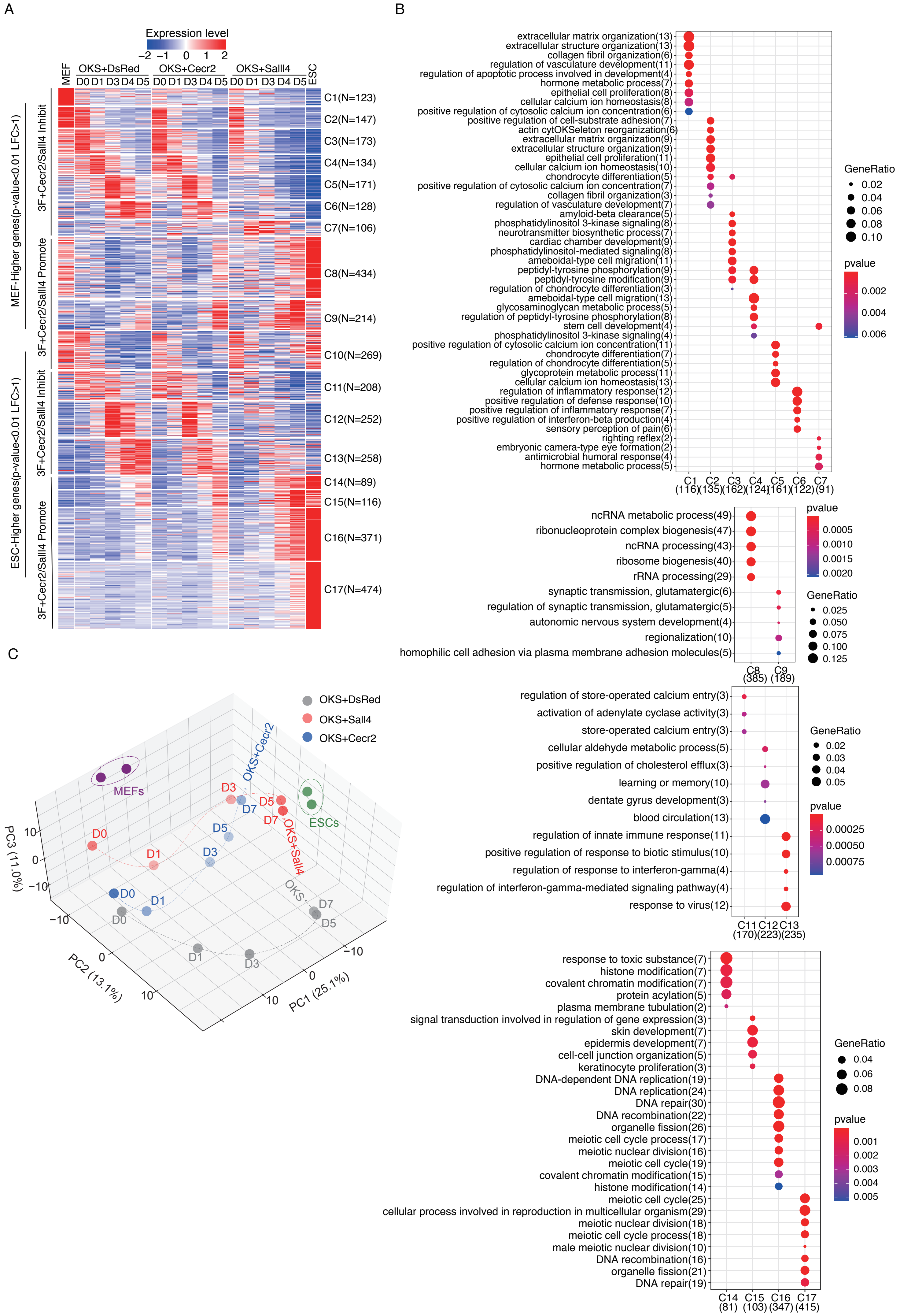

Supplementary Figure 4. Related to Figure 4.

The relationship between Cecr2 and Sall4 in reprogramming.

(A) Heatmaps for the RNA-seq data of MEF, OKS+Cecr2, OKS+Sall4 and ESC. MEFs infected with DsRed as Control.

(B) Gene Ontology (GO) analysis for genes in (A).

(C) ATAC-seq data shows similarities between OKS+Cecr2 and OKS+Sall4 reprogramming.

Supplementary Figure 5

A

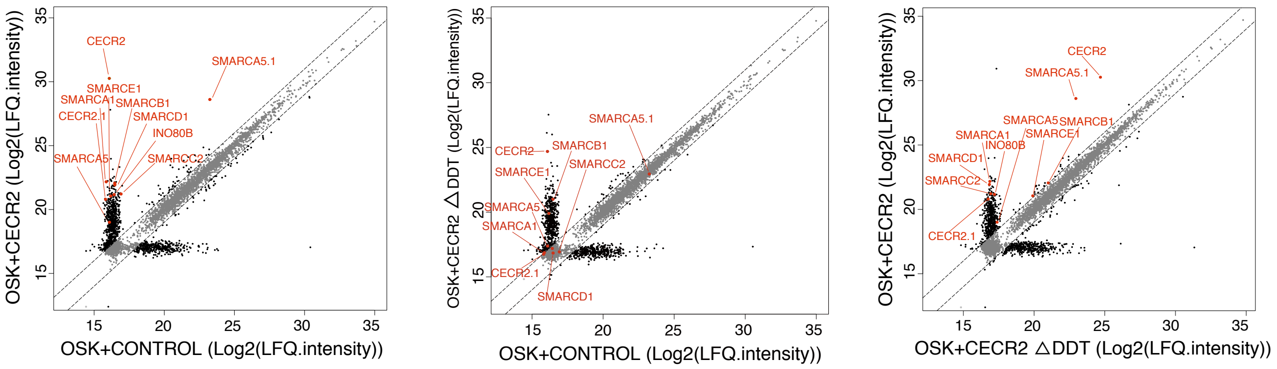

B

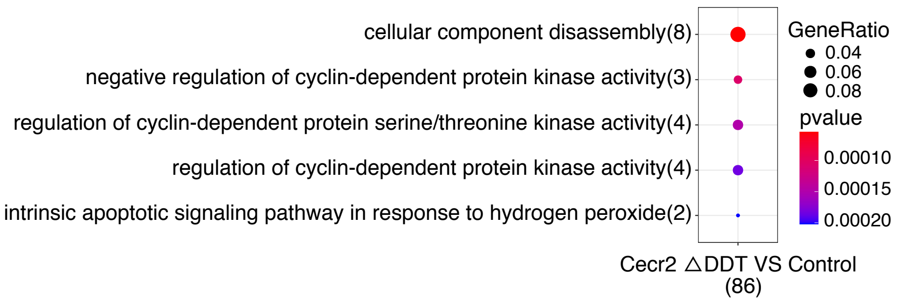

Supplementary Figure 5. Related to Figure 5.

The endogenous IP MS results showed that CECR2 interacted with SNF2L and other chromatin remolding related proteins.

(A) Scatter plot showed FLAG IP MS result of OKS+Cecr2-3×Flag compared to OKS+DsRed-3×Flag(Control), OKS+Cecr2 ΔDDT-3×Flag compared to OKS+DsRed-3×Flag(Control) and OKS+Cecr2-3×Flag compared to OKS+Cecr2 ΔDDT-3×Flag in day3 of reprogramming.

(B) GO analysis of top 100 proteins enriched in OKS+Cecr2 ΔDDT-3×Flag compared to OKS+DsRed-3×Flag(Control) reprogramming in day3 through FLAG-IP MS.

Supplementary Figure 6

A

Summary of Embryo Injection

Cecr2 $^{-/-}$ mESC	Injected Embryos	Recipient Females	Lived Embryos
Tetraploid	178	8	0
Chimera	159	9	10

B

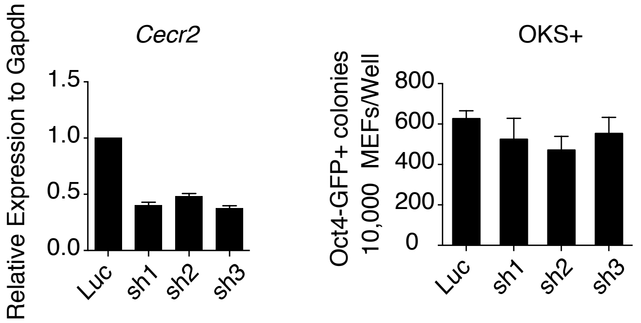

Supplementary Figure 6. Related to Figure 6.

The knockdown of Cecr2 in OKS induced reprogramming.

(A) Table summarizing Cecr2 knockout(KO) MEFs produced by blastocyst injection with Cecr2 knockout mESCs.

The knockdown of Cecr2 by shRNA had little effect on OKS reprogramming.
